# Exploring the Surface of the Ectodomain of the PD-L1 Immune Checkpoint with Small-Molecule Fragments

**DOI:** 10.1021/acschembio.2c00583

**Published:** 2022-09-08

**Authors:** Radoslaw Kitel, Ismael Rodríguez, Xabier del Corte, Jack Atmaj, Magdalena Żarnik, Ewa Surmiak, Damian Muszak, Katarzyna Magiera-Mularz, Grzegorz M. Popowicz, Tad A. Holak, Bogdan Musielak

**Affiliations:** † Faculty of Chemistry, Organic Chemistry Department, Jagiellonian University, Gronostajowa 2, 30-387 Krakow, Poland; ‡ Departamento de Química Orgánica I, Centro de Investigación y Estudios Avanzados “Lucio Lascaray” − Facultad de Farmacia, University of the Basque Country, UPV/EHU Paseo de la Universidad 7, 01006 Vitoria-Gasteiz, Spain; § Institute of Structural Biology, Helmholtz Zentrum München, Ingolstädter Landstrasse 1, 85764 Neuherberg, Germany

## Abstract

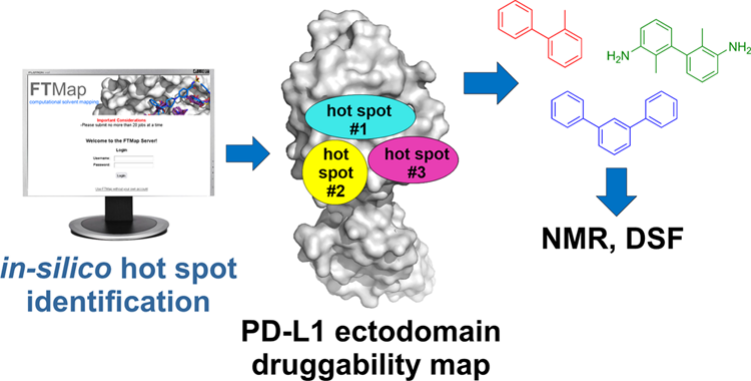

Development of small molecules targeting the PD-L1/PD-1 interface is advancing both in industry and academia, but only a few have reached early-stage clinical trials. Here, we take a closer look at the general druggability of PD-L1 using *in silico* hot spot mapping and nuclear magnetic resonance (NMR)-based characterization. We found that the conformational elasticity of the PD-L1 surface strongly influences the formation of hot spots. We deconstructed several generations of known inhibitors into fragments and examined their binding properties using differential scanning fluorimetry (DSF) and protein-based nuclear magnetic resonance (NMR). These biophysical analyses showed that not all fragments bind to the PD-L1 ectodomain despite having the biphenyl scaffold. Although most of the binding fragments induced PD-L1 oligomerization, two compounds, TAH35 and TAH36, retain the monomeric state of proteins upon binding. Additionally, the presence of the entire ectodomain did not affect the binding of the hit compounds and dimerization of PD-L1. The data demonstrated here provide important information on the PD-L1 druggability and the structure–activity relationship of the biphenyl core moiety and therefore may aid in the design of novel inhibitors and focused fragment libraries for PD-L1.

## 1 Introduction

Cancer immunotherapy, the process of mobilizing the immune system to fight cancer, represents an emerging approach in treating even advanced tumors. Taking into account the spectacular success of anti-programmed cell death protein 1 (anti-PD-1) or anti-programmed death ligand 1 (anti-PD-L1) monoclonal antibodies and their clinical outcomes, it seems that cancer immunotherapy may soon become the first-line treatment for a broad spectrum of tumors.^1,2^ There are multiple immune checkpoints that regulate the activity of the immune system against cancer cells. Among them, the PD-L1/PD-1 axis seems to play a central role in cancer immune surveillance. The number of FDA-approved antibodies targeting either PD-1 or PD-L1 reached seven agents and far more are now undergoing clinical trials.^3^ This clinical development of the antibodies targeting PD-L1 or PD-1 is in stark contrast with the progression of small molecules into the clinic. Although a myriad of compounds have been developed by pharmaceutical companies and in academia, until today, there are only five compounds that reached phase I or II of clinical trials.^4−8^

The PD-L1/PD-1 interface represents a typical protein–protein interaction (PPI), where both partners bind through large and flat surfaces. Historically, such PPIs are difficult to target with small molecules.^9^ Notwithstanding, such PPIs could be targeted with small molecules through identification of hot spots, which are the regions of protein surfaces that contribute disproportionally high to the binding energy.^10,11^

In recent years, fragment-based screening has been successfully applied in the development of potent inhibitors against multiple targets, including protein–protein interactions (PPIs).^12−14^ This yielded more than 40 small molecules discovered using this technique that have entered clinical trials, and five of them were approved by FDA.^15,16^ Apart from this, fragment-based approaches serve as methods for identification of hot spots on proteins, thereby allowing to determine their druggability. Moreover, fragment-based drug discovery (FBDD) approaches may also help in the identification of so-called cryptic sites on the surface of proteins. The primary obstacle in the identification of hot spots with fragments lies in the application of an appropriate method that allows the identification of weakly binding compounds. The development of multiple biophysical methods in the last two decades resulted in a large repertoire of techniques that are now available for FBDD.^17^ Nonetheless, the method of choice in FBDD is nuclear magnetic resonance (NMR) that is able to detect even weak interactions between proteins and fragments. This is of particular interest in FBDD because of the low affinity of initial fragments (typically in the mM range).^18,19^

To complement the spectrum of experimental methods, a large number of computational techniques were developed recently.^20−22^ For some of them, identification of hot spots was able to recapitulate the original primary binding site, highlighting the robustness of *in silico* methods.

In previous reports, we carried out systematic deconstruction of one of the first generations of the PD-1/PD-L1 compounds developed by Bristol–Myers Squibb (BMS).^23,24^ The individual fragments were then screened to study their binding mode to PD-L1 using two-dimensional NMR. These experiments revealed that the minimal fragment that binds to PD-L1 is represented by the biphenyl structural motif. In fact, the biphenyl core is present in all compounds targeting PD-L1 that has been developed so far. This not only implicates that this fragment serves as a driving portion in binding to PD-L1 but also suggests an existence of an important hot spot on the surface of PD-L1, where the biphenyl core is located.

Taking this into account, here, we probed computationally the surface of PD-L1 to determine its druggability and identify potential additional hot spots that have not been found previously.^25^ We confirmed the presence of the primary hot spot in the apo-protein and BMS-like-compound-bounded structures and noticed that it differs from that one, which is present in the PD-L1/PD-1 complex or PD-L1 bound to macrocyclic peptides.^26^ In such a way, we identified additional two secondary hot spots that lay in close proximity to the major one. However, we conclude that only one of them could be used for the extension of BMS-like molecules. Finally, we assembled a set of 38 fragments containing the biphenyl motif that was extracted from two generations of inhibitors that have been developed so far. This resulted in a focused fragment library that has been subsequently used in a typical biophysical triage by differential scanning fluorimetry (DSF) and NMR (NMR) spectroscopy. In contrast to our previous reports, here, we used the entire ectodomain of PD-L1 to check whether the oligomerization, a previously recognized phenomenon for BMS-like inhibitors, depended on the presence of the C2 domain. We found that not all biphenyl fragments were able to bind to the PD-L1 surface and the binding strongly depended on the substitution pattern of the biphenyl core moiety. Moreover, we conclude that the C2-type domain of PD-L1 did not play a role in the compound binding mode of the PD-L1 ectodomain. Taken together, our results provide important information on the druggability of the PD-L1/PD-1 interface and the structure–activity relationships of the biphenyl fragments.

## 2 Results and Discussion

### 2.1 *In Silico* Analysis of PD-L1 Druggability

We first considered an *in silico* approach to test the druggability of the PD-L1 surface using the FTMap server.^27^ FTMap docks 16 small molecular probes that differ in size, shape, and polarity (Table S2) onto the protein surfaces to identify the druggable hot spots. The principal hot spot is defined as a consensus site (CS) containing the highest number of probe clusters.

**Figure 1:**
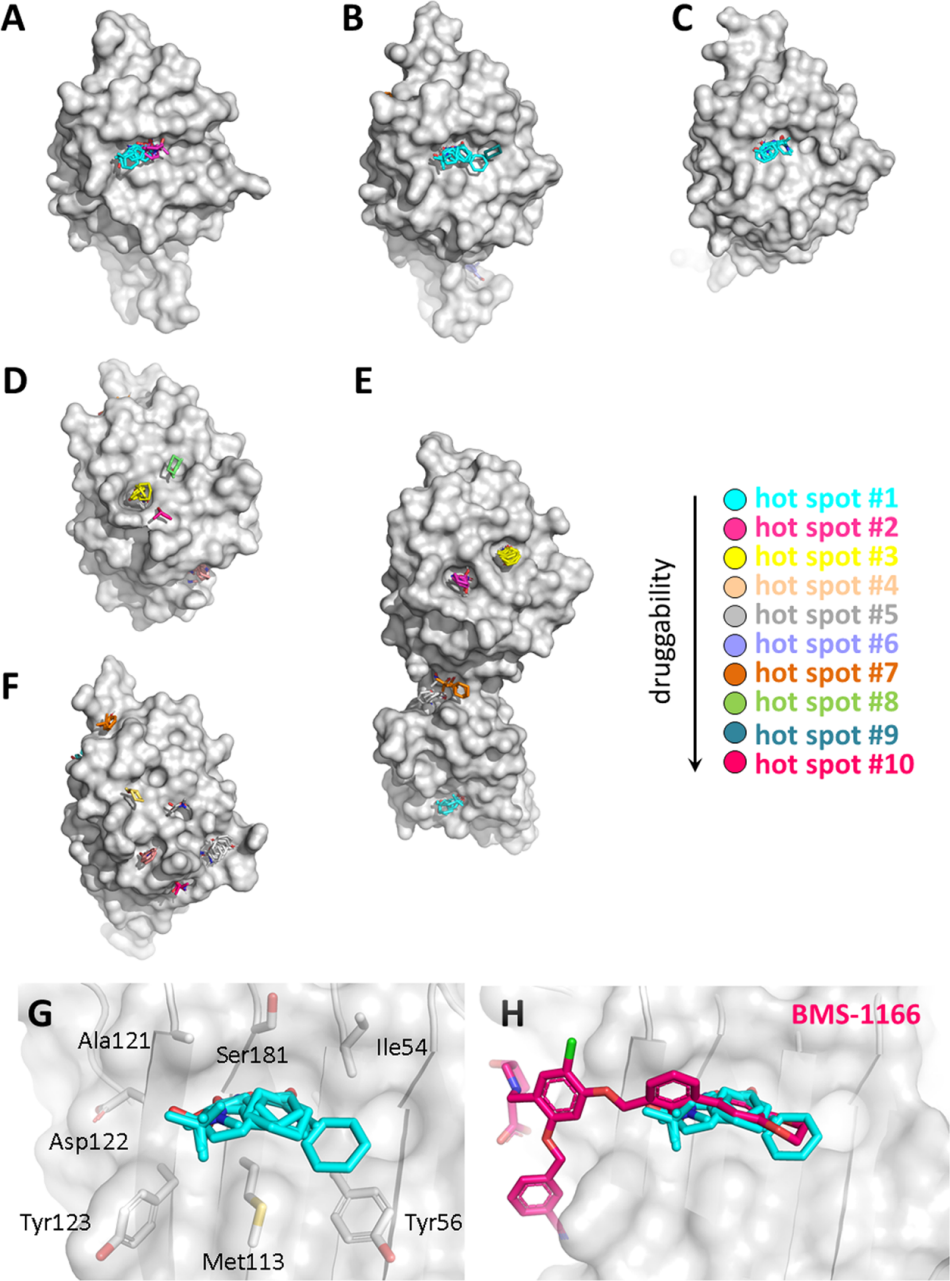
*In silico* surface probing of PD-L1 with FTMap; (A–F) localization of predicted hot spots on the surface of six X-ray structures of PD-L1, the primary hot spot is represented by probes colored with cyan, (G) architecture of primary hot spots in the BMS-1166-PD-L1 complex, and (H) mapping the main hot spot with the original location of **BMS-1166**; the hot spot ranking is also shown.

Given the high level of flexibility of the PD-L1 surface upon binding either PD-1 or small molecules, we aimed to identify hot spots on five different human and one mouse PD-L1 X-ray structures to take into account the effects of conformational rearrangements and differences between species. Initially, we applied this analysis using standard parameters to apo-PD-L1 (PDB: 5C3T) and two PD-L1 structures extracted from the complexes with the small-molecule inhibitors, **BMS-1166** and compound A (**cmpd A**) (PDB: 6R3K and 6VQN, respectively).^28^ Furthermore, we also use the X-ray structure of PD-L1 from the complex with PD-1 (PDB: 4ZQK), PD-L1 ectodomain co-crystalized with a macrocyclic peptide (PDB: 6PV9), and mouse PD-L1 (PDB: 6SRU). The location of the identified hot spots on the surface of PD-L1 is shown in Figure 1A–F. Each consensus site is represented by different probe colors, and the ranking of hot spots is shown in Figure 1. The detailed characteristics of the primary hot spots are listed in Table 1. In the interpretation of the results, we followed the general classification of protein druggability and hot spot characteristics for FTMap published elsewhere.^11^ Accordingly, we labeled the hot spot primary when it contains the highest number of probe clusters.

**Table 1:** Summary of Primary Hot Spot Analysis using FTMap

		primary hot spot no. 1		
structure	resolution (Å)	Ig domain	PD-L1/PD-1 interface	no. of probe clusters
5C3T	1.80	V1	yes	15
6R3K	2.20	V1	yes	22
6VQN	2.49	V1	yes	23
4ZQK	2.45	V1	no	19
6PV9	2.00	C2	no	17
6SRU	2.53	V1	no	14

Interestingly, we observed different locations of primary hot spots depending on the X-ray structure used in the analysis. In the case of apo-PD-L1 (Figure 1A) and PD-L1 from complexes with **BMS-1166** (Figure 1B) and **cmpd A** (Figure 1C), the primary hot spot is located in the same position on the protein surface and lies on the PD-L1/PD-1 interface. This hot spot contains 15, 22, and 23 probe clusters in apo-PD-L1, BMS-1166-PD-L1, and cmpd A-PD-L1 structures, respectively. In contrast, the FTMap analysis of the surface of PD-L1 in its complex with PD-1 (Figure 1D) or a macrocyclic peptide (Figure 1E) suggests the presence of hot spots with lower druggability scores (secondary hot spots) and is located in a different region of the PD-L1/PD-1 interface. The common main hot spot in these two structures lies in a cleft formed by the side chains of Tyr123 and Arg113. However, this hot spot is not evident anymore upon binding of BMS-like small molecules. This is related to the flexibility of some amino acid side chains that change their conformation upon binding of small molecules. In particular, the side chain of Met115 plays an important role in the formation of hot spots. We mined available crystal structures of PD-L1 in complexes with biphenyl-based small molecules from PDB, and we noticed that binding of such small molecules always induced bending of the side chain of Met115, thereby filling the pocket formed by Tyr123 and Arg113, recognized here as the main secondary hot spot. This results in closing the pocket and making targeting this hot spot impossible when starting elaboration of an inhibitor from a biphenyl fragment.

Finally, no primary hot spots were detected on the surface of mouse PD-L1 (Figure 1F). This is in perfect agreement with recent findings that mouse PD-L1 does not interact with BMS-like inhibitors.^29^

The primary strong hot spot identified in three structures (Figure 1A–C) is formed by the side chains of Ile54, Tyr56, Met115, Ser117, and Ala121 and partially Tyr123. Additionally, backbone atoms of Val55, Ile116, and Asp122 help to form the entire hot spot (Figure 1G). This hot spot is precisely located on the site of the biphenyl moiety from BMS-like compounds (Figure 1H). Interestingly, apart from the structures of the PD-L1 complexes with **BMS-1166** and **cmpd A**, the FTMap was also successful in locating the primary hot spot precisely in the binding site of the biphenyl scaffold of BMS-like compounds on the surface of apo-PD-L1. This confirms previous observations on other proteins that even an unliganded structure is sufficient for proper identification of the primary hot spot.^30^ Additionally, this shows that the FTMap analysis is not dependent on the original ligand location since the last one is removed before calculations.^11^

In summary, the FTMap-based analysis of druggability carried out on six different X-ray structures of PD-L1 suggests that the main hot spot is located on the interface of PD-L1/PD-1. However, depending on the PD-L1 conformational state, the primary hot spots were located in the different regions of the interface. Notably, the presence of one of the secondary hot spots depends strongly on the binding of biphenyl fragments.

### 2.2 Biphenyl Focused Library Design

**Figure 2:**
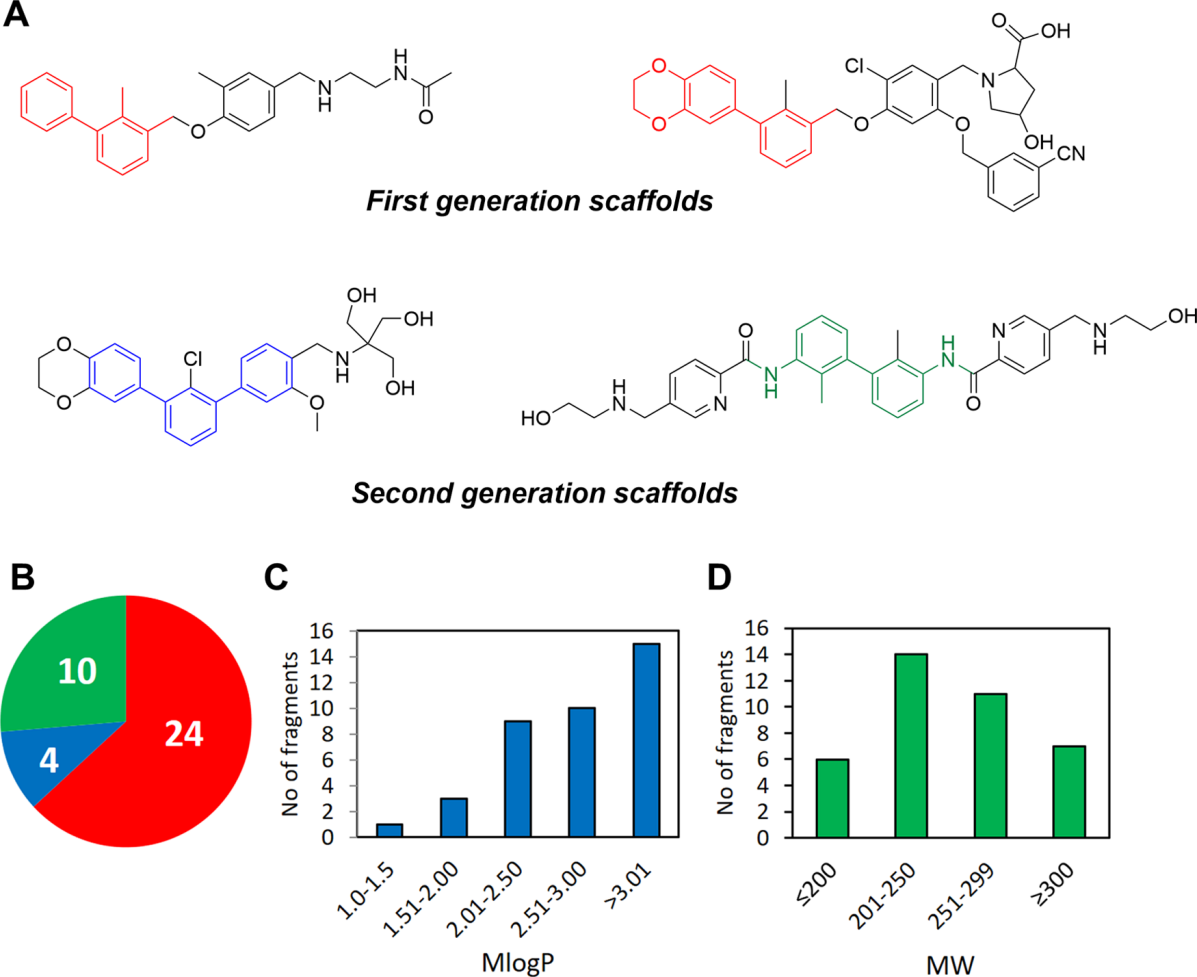
PD-L1 focused fragment library. (A) Structures of known PD-L1 inhibitors used for deconstruction of biphenyl fragments; each subset contains substituted fragments with an overall structure highlighted in red (first generation scaffolds), blue, and green (second generation scaffold). (B) Distribution of fragments in each subset. (C) Distribution of fragments according to calculated *M* log *P* values. (D) Distribution of fragments according to molecular weight (MW).

Taking into account the perfect overlap of the biphenyl fragment from **BMS-1166** and **cmpd A** with the predicted primary hot spot, we further examined how variations of the substituents in the biphenyl will affect the binding. To this end, we created small but diverse focused library of fragments containing the biphenyl core. Our library was designed based on the visual inspection and available data on the binding potency of three main classes of the published PD-L1 inhibitors (Figure 2A). The library contains three subsets of fragments derived from different generations of PD-L1 inhibitors. The first class represents fragments from the earliest generation of PD-L1 inhibitors, and the second one is populated with terphenyl fragments, which are developed recently,^31,32^ and also contains fragments from symmetrical and elongated compounds that represent the latest approach in the design of PD-L1 inhibitors. The overall structures of fragments are highlighted in red (first class), blue and green for the second class. In total, our library contains 38 fragments that differ in the substitution pattern of the biphenyl or terphenyl core (Figure 2B). Taking into account the lipophilic nature of biphenyls and terphenyls, 39% of the fragments within the library have *M* log *P* values above 3 with a maximum value of 4.365. In terms of molecular weight, only 18% of compounds do not adhere to a widely accepted value of 300 with a maximum value of 377 Da. The remaining compounds within the library meet the criteria accepted for fragments in terms of molecular weight and partition coefficient described by Kirsch and co-workers with average MW 256 and *M* log *P* 2.93.^33^ The distribution of fragments according to their molecular weight and *M* log *P* values is shown in Figure 2D.

### 2.3 Expression and Purification of PD-L1

**Figure 3:**
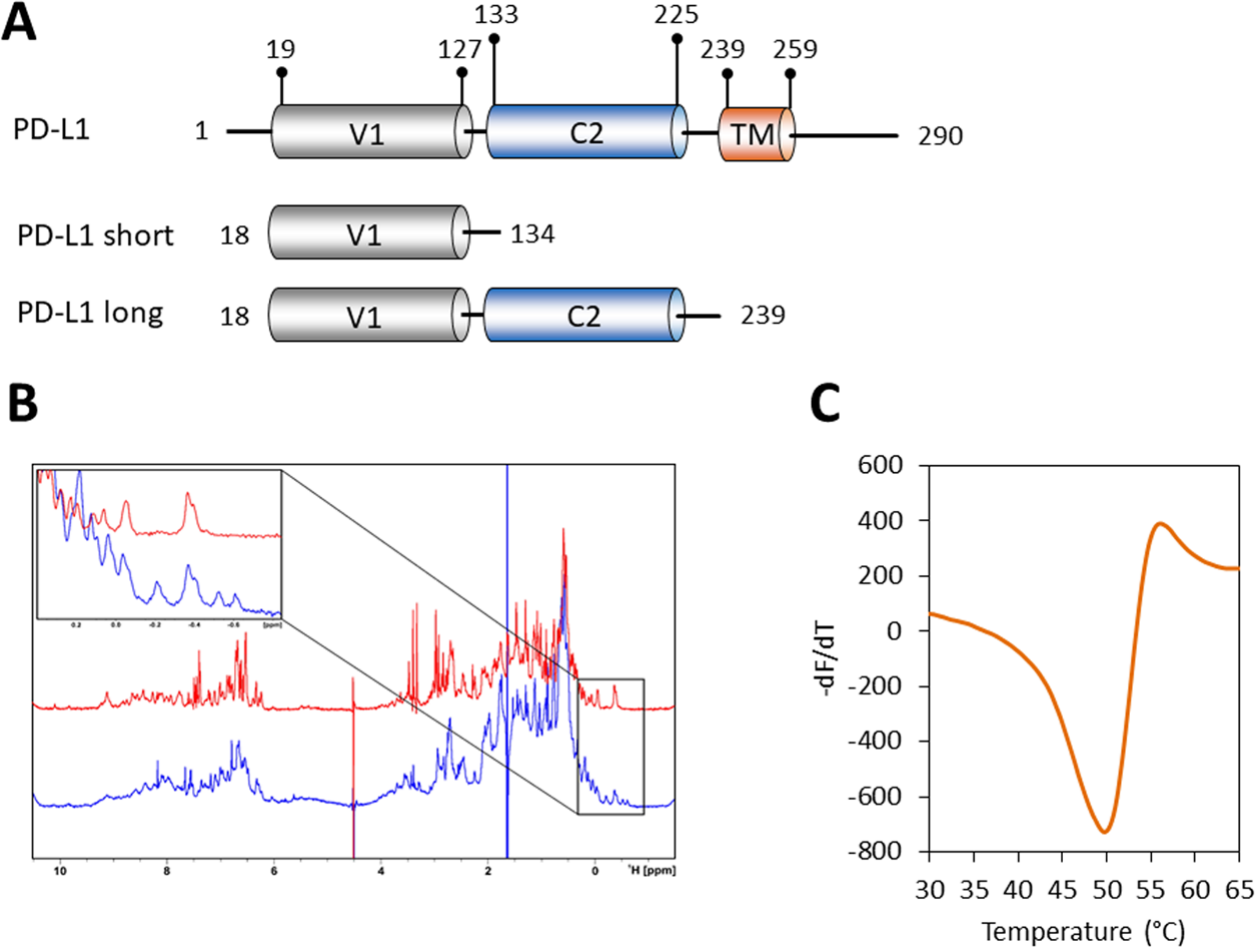
Characteristics of PD-L1 constructs. (A) Domain organization of full-length human PD-L1 (UniProt accession code: Q9NZQ7) and its truncations used in the study. (B) ^1^H NMR spectra of apo-PD-L1-long (blue) and apo-PD-L1-short (red) with an enlarged aliphatic region. (C) Melting curve of PD-L1-long.

Human PD-L1 (hPD-L1) contains 290 amino acids with short both transmembrane (TM) and cytoplasmic sequences (residues 239–259 and 260–290, respectively). The ectodomain of hPD-L1 (amino acids 19–238) contains two, the Ig-like V-type and Ig-like C2-type, ca. 100 amino acid domains separated by a short linker (Figure 3A). The first N-terminal domain (with the V-type fold) of hPD-L1 is responsible for binding to PD-1. The role of the C-terminal domain characterized by the Ig-like C2-type fold is unknown.

We successfully expressed and purified the entire ectodomain of PD-L1 (18–239). The protein was properly folded as verified by NMR and DSF experiments (Figure 3B,C). We noticed that the aliphatic region of the NMR spectrum contains well-dispersed resonances that should allow detecting the binding events (Figure 3B).

### 2.4 Fragment-Based Screening using a Focused Fragment Library

Having the library in hand, we carried out screening using differential scanning fluorimetry and 1D NMR techniques. Previously, the fragment-based approach with a large library of compounds (>13 000) was tested on the PD-L1 V1 domain.^33^ This, however, led to the identification of compounds bearing similar structural features to those in the BMS-like molecules.^34^ This confirms that the biphenyl core of the BMS-like inhibitors serves as a driving force of the compounds that bind to PD-L1. Therefore, instead of using a large fragment library, we assembled a focused library of fragments containing the biphenyl core extracted from known inhibitors. The obtained fragments were decorated with different substituents to test how these variations will affect the binding of biphenyl.

Fragments were screened in both techniques as singletons. The detailed results are summarized in Table S1. The melting point of the PD-L1 ectodomain measured in DSF was 49.6 °C and was only slightly affected by the presence of 2% DMSO (49.2 °C). The fragments were tested at a final concentration of 0.1 mM. As a positive control, **BMS-1166** was used, which stabilizes the PD-L1 ectodomain by 2.7 °C when tested at 0.1 mM. DSF hits were defined as fragments, resulting in an increase or decrease in melting point of PD-L1 by at least 0.5 °C. Based on this threshold value, 15 compounds were identified as hits, corresponding to a hit rate of 39%. This hit rate includes both true and false positive hits and, as expected, is much higher than for random fragment library. The larger recorded positive Δ*T*_m_ was for fragment **TAH47**, which stabilized the PD-L1 ectodomain by 1.3 °C. On the other hand, **TAH36** appeared to be the strongest destabilizing fragment with a Δ*T*_m_ value −1.4 °C (Table 1). Interestingly, fragments **TAH35** and **TAH36** derived from one of the most potent inhibitors discovered so far, **cmpd A**, had a destabilization effect on PD-L1. This confirms that fragments that initially destabilize the protein could be turned into potent strong inhibitors.

In parallel, we carried out a series of one-dimensional ^1^H NMR spectroscopy binding experiments. In these assays, binding was assessed by comparing the spectra collected in the presence of DMSO with those in the presence of fragments. All fragments were tested at a protein/ligand molar ratio of 1:10. Fragments were considered as hits in the NMR screen if they showed clear chemical shift perturbations and/or broadening of the resonances in the aliphatic region of the spectrum. The detailed results on compound binding are provided in Table S1.

Interestingly, NMR-based screening resulted in 25 compounds that were flagged as hits, which accounts for the overall remarkable hit rate of 63%. This highlights the advantage of NMR over other biophysical techniques, in which the weak binders are very often omitted. In summary, 14 fragments were identified in both applied techniques.

Although all compounds within our in-house library contain the biphenyl core, not all of them were identified as binders (Table 1). The substitution pattern of biphenyl apparently dictates the potency of the fragment, and even small structural changes within the particular class were beneficial or deleterious for binding. It is evident that the amine group in the position at C2 of the right-handed ring of biphenyl (or central ring in the case of terphenyl fragments) is responsible for activity dropping. Nonetheless, some fragments, namely, **TAH44**, **TAH53**, **TAH54**, **TAH57**, **TAH58**, and **TAH59**, having amine at this position were still active. This could be attributed to slight changes in fragment orientation on the protein surface compared to the elaborated molecule. Such a situation, when the fragment does not recapitulate the original orientation found in the optimized ligand, has been previously observed in compound deconstruction approaches.^35^

**Figure 4:**
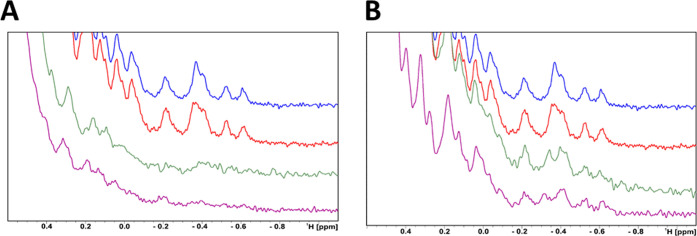
Results of NMR-based validation of selected fragment hits on the PD-L1 ectodomain. (A) Fragments triggering dimerization of PD-L1 ectodomain–^1^H NMR spectra of apo-PD-L1-long (blue) and with DMSO-*d*_6_ (red), **TAH4** (green), and **TAH47** (purple). (B) Fragments that do not induced PD-L1 ectodomain dimerization–^1^H NMR spectra of PD-L1-long with **TAH35** (green) and **STD4** (purple).

One of the additional advantages of NMR spectroscopy is that it provides insights into the oligomerization state of the protein in solution in the absence or presence of compounds. In fact, all BMS-like inhibitors induce PD-L1 homodimerization upon binding,^36^ and therefore, NMR spectroscopy is an ideal method for detecting such events. We and others showed previously that the biphenyl motif serves as a driving force in homodimerization of PD-L1.^23,34^ This was also valid for most of the active fragments within this study, which confirms that the biphenyl and terphenyl motifs are responsible for PD-L1 dimerization (Figure 4A). Of note, some active biphenyl fragments extracted from the symmetric small-molecule **cmpd A**: **TAH35** and **TAH36**, did not induce PD-L1 dimerization (Figure 4B). Interestingly, these fragments displayed the most negative Δ*T*_m_ values in the DSF-based screen. The behavior of these fragments was similar to compound **STD4**, discovered by us in an NMR-based FBDD campaign recently (data not shown) (Table S1 andFigures S1 and S2), a weak binder that displayed *K*_D_ = 2.21 mM and did not induce oligomerization of PD-L1.

**Figure 5:**
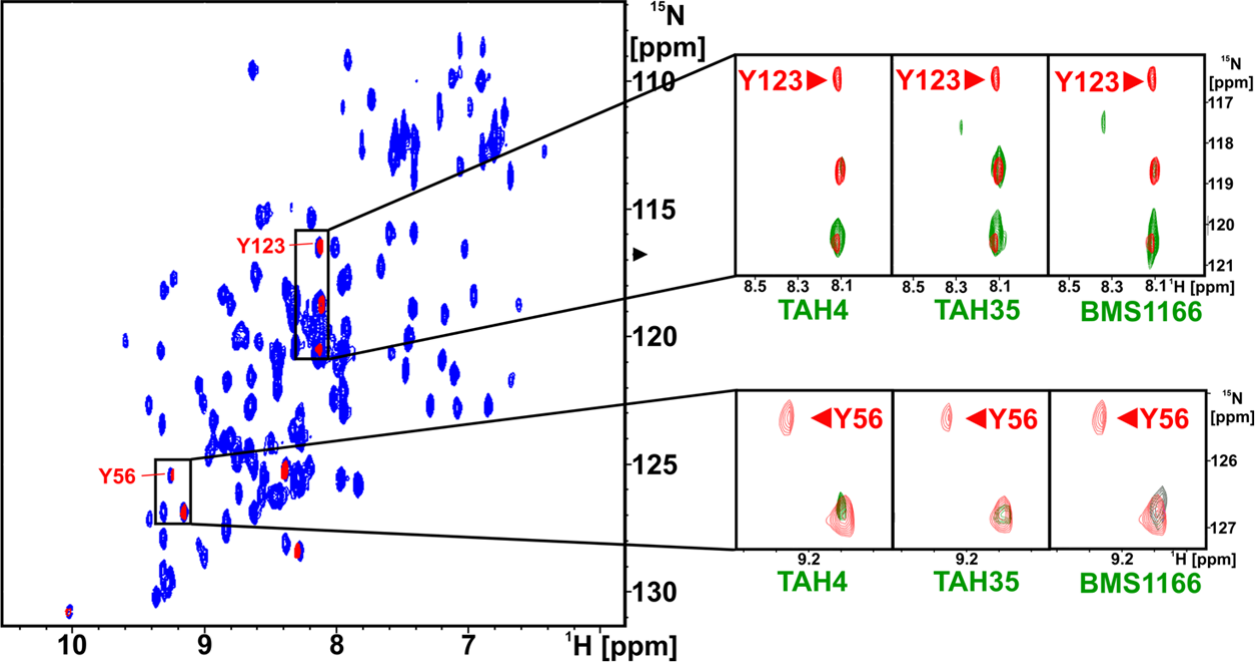
^1^H–^15^N HMQC NMR spectra of apo-PD-L1 (blue) and selective labeling of tyrosine in apo-PD-L1 (red) with marked Tyr56 and Tyr123 residues, which form the hydrophobic pocket of PD-L1. In enlarged parts of the spectra, the critical residues’ (Tyr56 and Tyr123) behavior for interactions between PD-L1 and inhibitors is shown (green). In all cases, significant broadening of the signals (disappearance) or their shift is observed under the influence of the tested inhibitors (**TAH4** and **TAH35**) and **BMS-1166** as a positive control. For the remaining tyrosine, which is not located in the binding pocket, no signal changes are observed.

Additionally, to test whether the fragments bind to the primary hot spot, we carried out co-crystallization experiments of fragments **TAH4** and **TAH35** with the PD-L1 ectodomain, however, we failed to obtain diffraction quality crystals. To overcome this, we run an NMR experiment with PD-L1 (short) labeled selectively on tyrosine residues^37^ (Figure 5). The primary hot spot is located between Tyr56 and Tyr123, and therefore, any binding events in this area should be detectable by perturbation of corresponding cross peaks. The addition of **TAH4** resulted in disappearance of Tyr123 and Tyr56 cross peaks, which confirms its binding to the primary hot spot. On the other hand, both **TAH35** and **BMS-1166** induced a shift of Tyr123 cross peak and additionally disappearance of the Tyr56 signal. Together, these data confirm that the tested fragments bind to the primary hot spot.

**Figure 6:**
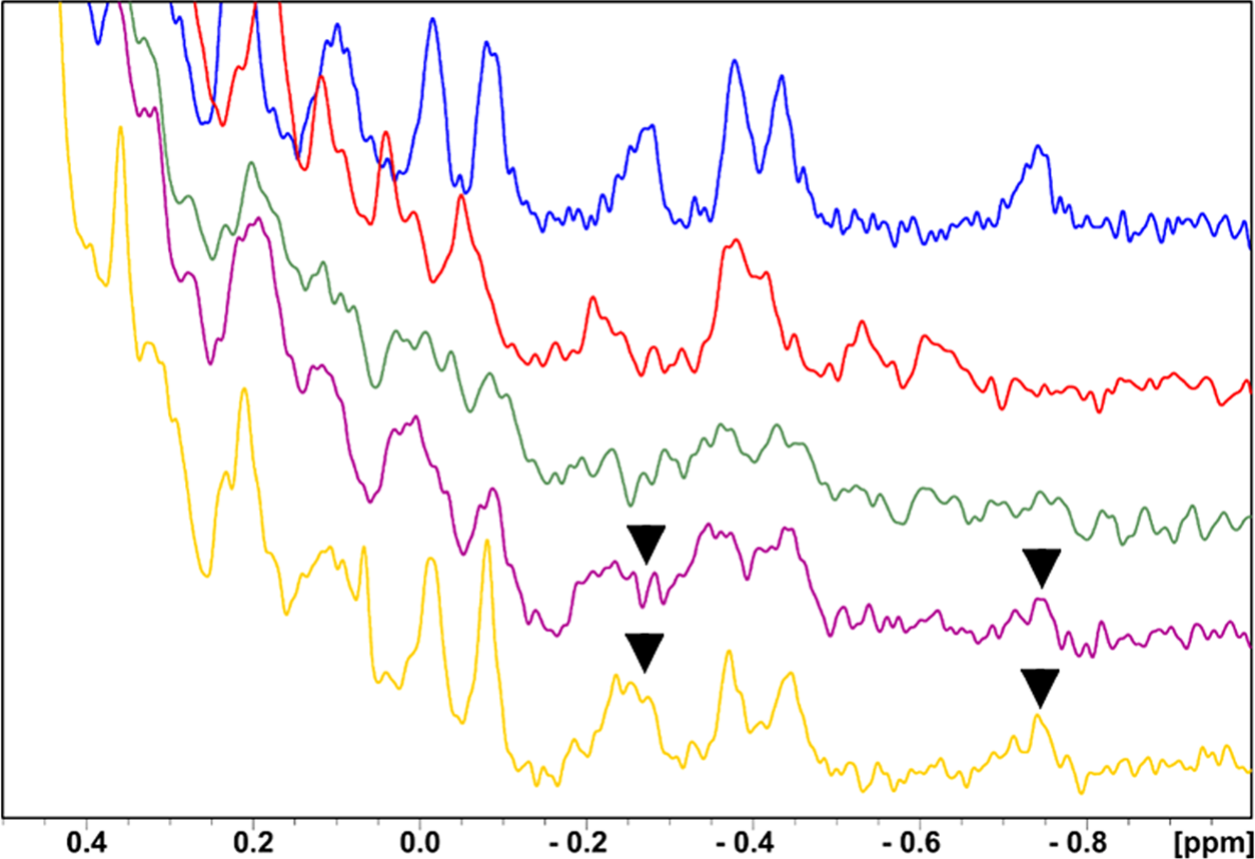
Aliphatic part of ^1^H NMR spectra of PD-1 (blue), long-PD-L1 (red), the complex of PD-1/long-PD-L1 (green). The complex of PD-1/long-PD-L1 with **TAH35** (purple) in the molar ratio protein to the compound 1:20, respectively, and the complex of PD-1/long-PD-L1 with **BMS-1166** (as a positive control to show that the complex can be dissociated), the molar ratio 1:1 of the protein and the compound (yellow). Arrows indicate restoring of PD-1 signals.

Finally, to estimate the *K*_D_ value between **TAH4/TAH35** and long-PD-L1, we carried out a 1D w-AIDA-NMR experiment (w-AIDA-NMR: weak-antagonist induced dissociation assay-NMR) (Figure 6).^38,39^ We used unlabeled proteins PD-1 (13.2 kDa) and long-PD-L1 (24.3 kDa). After addition of long-PD-L1 to PD-1 (in the molar ratio of 1:1), most of the signals in the proton spectrum of PD-1 became broader and their intensities decreased.^38,40,41^ Noticeable changes in the chemical shifts could be observed in the range ca. 0.4 to −1.0 ppm. This result confirms the formation of the complex with the molecular weight ca. 37 kDa. The w-AIDA-NMR assay was then applied to test the dissociating capabilities of **TAH35** and **TAH4**. We could estimate the dissociation constant of the long-PD-L1/fragment **TAH35** interaction,^42^ which was in the range of 20 ± 10 mM. In the case of fragment **TAH4**, for which the w-AIDA-NMR indicated less recovery of the NMR signals; we determined that the *K*_D_ was around 40 mM (data not shown). Full recovery of PD-1 signals was observed only after addition of **BMS-1166** (Figure 6).

It remains to be confirmed whether homodimerization as a primary mechanism of action of PD-L1 inhibitors is sufficient to prevent its interaction with PD-1 under *in vivo* conditions. Our study demonstrates that the entire PD-L1 ectodomain, which is a surrogate of a native protein on the cell membrane, is able to dimerize in solution, and therefore, this mechanism of action might also be valid in the cell environment.

## 3 Conclusions

In this work, we looked for identification of hot spots on the PD-L1 surface to assess its druggability and found additional potential pockets that could be targeted to increase the potency of available inhibitors. As a result, we provided here a comprehensive analysis and a detailed map of hot spots that are druggable with either small molecules or macrocyclic peptides. This should provide general guidance for a rational design of PD-L1/PD-1 inhibitors that combines the structural features of BMS-like compounds and macrocyclic peptides.

We also screened a small library of fragments derived from well-known biphenyl-based inhibitors against the whole ectodomain of PD-L1. We confirmed here that the PD-L1 ectodomain is able to dimerize in the presence of active fragments, indicating that this mechanism of action is also plausible in the cell environment. Altogether, we believe that the detailed hot spot map of the PD-L1 surface described here will bolster efforts to optimize the current inhibitors and to develop other chemical series.

## 4 Methods

### 4.1 FTMap Analysis

The FTMap server (http://ftmap.bu.edu) was used for detection of hot spots on the PD-L1 surface. The following holo X-ray structures were used: 4ZQK, 6R3K, 6VQN, and 6PV9. As apo, we used 5C3T. In each case, only chain A was taken for analysis. Before uploading, ligands and water molecules were removed. Upon completion, the results were downloaded and inspected, and the location of probe clusters was visualized in PyMol software.^43^ For data interpretation, we followed the recommendations published elsewhere.^11^

### 4.2 PD-L1 Expression and Purification

The expression and purification protocol relate to work on PD-L1-short published earlier by us. Briefly, *Escherichia coli* strain BL21 (DE3) was transformed with a pET-21b plasmid carrying the PD-L1-long gene (amino acids 18–239). The bacteria were cultured in LB at 37 °C until OD_600_ _nm_ of 0.8 when the recombinant protein production was induced with 1 mM IPTG. The protein expression was carried out at 37 °C for 5 h. Next, bacteria cells were harvested, and the pellet was frozen at −20 °C. SDS-PAGE analysis revealed that the protein was expressed exclusively in the form of inclusion bodies. Inclusion bodies were collected by centrifugation, suspended in 1× PBS, and then sonicated to finally collect them again by centrifugation. Then, the inclusion bodies were washed twice with 50 mM Tris-HCl, pH 8.0, containing 200 mM NaCl, 10 mM EDTA, 10 mM 2- mercaptoethanol, and 0.5% Triton X-100, followed by a single wash with the same buffer without Triton X-100.

The washed inclusion bodies were resuspended overnight in 50 mM Tris-HCl, pH 8.0, 6 M Gunidine·HCl, 200 mM NaCl, and 10 mM 2-mercaptoethanol and clarified with centrifugation. Refolding of PD-L1 was performed by dropwise dilution into 0.1 M Tris-HCl, pH 8.0, containing 1 M l-arginine hydrochloride, 2 mM EDTA, 0.25 mM oxidized glutathione, and 0.25 mM reduced glutathione. The refolded protein was dialyzed three times against 10 mM Tris-HCl, pH 8.0, containing 20 mM NaCl and purified by size-exclusion chromatography using Superdex 75. For DSF measurements, the protein was filtrated in 20 mM HEPES, pH 7.5, 100 mM NaCl and for NMR measurements in 1x PBS. The quality of the refolded protein was evaluated by sodium dodecyl sulfate–polyacrylamide gel electrophoresis and NMR.

For ^15^N-labeled PD-L1-short, the following medium (1 L) was used: l-alanine, l-glutamine, l-glutamic acid, l-arginine (each 0.4 g/L), l-asparagine (0.255 g/L), l-methionine, cytosine, guanosine, uracil (0.125 g/L), l-aspartic acid, l-leucine, l-lysine, l-histidine, l-proline, l-threonine, l-glycine, l-isoleucine, l-valine, and ^15^N-l-tyrosine (0.1 g/L), l-serine (1.6 g/L), CaCl_2_ (0.01 g/L), sodium acetate (2 g/L), K_2_HPO_4_ (10 g/L), citric acid (1 g/L), trace element solution (1.3 mL/L), ferrous citrate (0.036 g/L), Zn-EDTA (1 mL/L), and NH_4_Cl (1 g/L). After autoclaving, the following solutions were added: glucose (25 mL of 20%/L), thiamine (0.56 mL/L), MgSO_4_ (2 mL of 1 M/L), l-cysteine, l-tryptophan, nicotinic acid (0.05 g/L), and biotin (0.1 mg/L). After producing the bacterial pellet, the methodology of refolding and purification of the protein was the same as described for the unlabeled protein.

### 4.3 DSF Screening

Thermal melting experiments were carried out using a CFX96TM real-time PCR machine (BioRad). Protein thermal unfolding was monitored by the increase in the fluorescence of the SYPRO orange dye. To perform DSF experiments, 20 μM PD-L1 long, 20× SYPRO orange dye (Thermo Fisher Scientific, U.K.), and 0.1 mM compound in 10 mM Tris-HCl, pH 8.0, containing 20 mM NaCl were added to 96-well polymerase chain reaction (PCR) plates with a final volume of 40 μL. Subsequently, the samples were heated in a PCR system from 25 to 95 °C at a rate of 0.4 °C/10 s. Fluorescence intensities were monitored with 492 nm excitation and 610 nm emission. Control wells were used to compare the melting temperature (*T*_m_) without fragments [replaced by the same amount of dimethyl sulfoxide (DMSO)] and with 0.1 mM **BMS-1166** as a positive control. *T*_m_ values were obtained from the maximum value of first derivative (d*F*/d*T*) plots of the unfolding protein curves and then analyzed in Microsoft Excel. Experiments were performed in triplicate. Thermal shift values (Δ*T*_m_) were obtained through subtraction of the unfolding temperature of the PD-L1 ectodomain in the presence of 2% (vol/vol) DMSO (T_mDMSO_) from unfolding temperatures of the PD-L1 ectodomain in the presence of fragment (*T*_mfr_), according to the following equation: Δ*T*_m_ [°C] = *T*_mfr_ – *T*_mDMSO_.

### 4.4 NMR Measurements

NMR measurements were carried out at 300 K on an ultra-shielded 600 MHz Bruker AVANCE III spectrometer equipped with a liquid nitrogen cryogenic system. To provide a lock signal, 10% (v/v) D_2_O was added to the samples. Typically, the PD-L1 ectodomain was used at a concentration of 0.15–0.2 mM. Fragments were tested at 10× molar excess with respect to proteins. The spectra were processed with TopSpin 3.2 software.

## Supporting Information Available

Results of the screening fragments with PD-L1 (DSF and NMR); NMR spectra of PD-L1 with **STD4**, **TAH35**, and **TAH36**; list of probes used in FTMap analysis (PDF)
