# Supplementary material for: Exploring the Surface of the Ectodomain of the PD-L1 Immune Checkpoint with Small-Molecule Fragments
Source: ACS Chem Biol. 2022 Sep 8;17(9):2655–63. doi: 10.1021/acschembio.2c00583 (PMC9486809; doi:10.1021/acschembio.2c00583)
Supplement: Supplementary file 1 — cb2c00583_si_001.pdf [file cb2c00583_si_001.pdf]

Supporting Information

Exploring the surface of the ectodomain of the PD-L1 immune checkpoint with small-molecule fragments

Radoslaw Kitel[a], Ismael Rodríguez[a], Xabier del Corte[b], Jack Atmaj[a], Magdalena Żarnik[a], Ewa Surmiak[a], Damian Muszak[a], Katarzyna Magiera-Mularz[a], Grzegorz M. Popowicz[c], Tad A. Holak[a], and Bogdan Musielak[a,*]

a - Faculty of Chemistry, Organic Chemistry Department, Jagiellonian University, Gronostajowa 2, 30-387, Krakow, Poland

b - Departamento de Química Orgánica I, Centro de Investigación y Estudios Avanzados "Lucio Lascaray"-Facultad de Farmacia, University of the Basque Country, UPV/EHU Paseo de la Universidad 7, 01006 Vitoria-Gasteiz, Spain

c - Institute of Structural Biology, Helmholtz Zentrum München, Ingolstädter Landstrasse 1, 85764, Neuherberg, Germany

*Email: bogdan.musielak@uj.edu.pl

Contents:

1. Table S1 – Results of screening S3

2. Figure S1 - STD-4 binds to short and long PD-L1 …………………………………. S9

3. Figure S2. STD4, TAH35 and TAH36 bind to PD-L1 ectodomain ………….……. S10

4. Table S2 - List of probes used in FTMap analysis… ………………..…………….. S11

Table S1. Results of the screening

Cmpd #	Structure	MW[a]	MLogP[a]	Binding assessment	
				DSF ΔT_m (°C)	1D NMR
TAH2	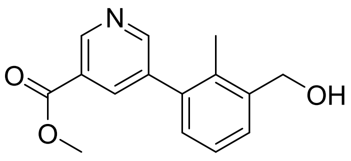	257.3	1.328	0.1	no
TAH3	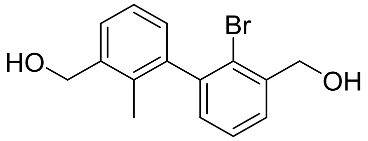	307.2	3.409	0.3	no
TAH4	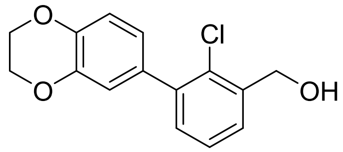	276.7	2.595	0.5	yes
TAH5	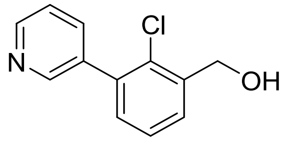	219.7	2.126	0.3	yes
TAH6	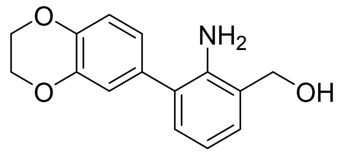	257.3	2.049	0.3	no

TAH7	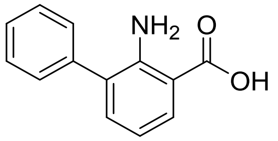	213.0	1.68	0.3	no
TAH8	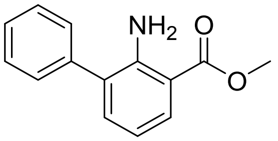	227.3	3.051	0.3	no
TAH9	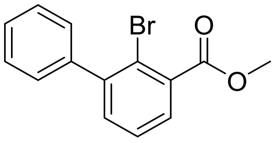	291.1	3.757	0.5	yes
TAH11	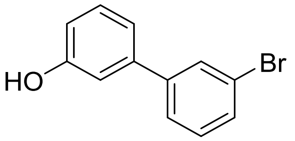	249.1	3.8	0.1	yes
TAH14	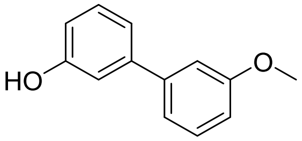	200.2	2.786	0.5	yes
TAH16	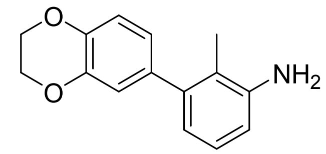	241.3	2.346	0.5	yes
TAH17	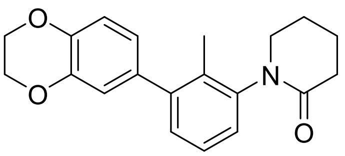	323.4	3.054	0.3	yes
TAH19	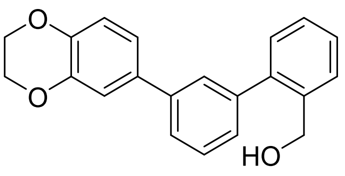	318.4	3.258	0.5	yes

TAH28	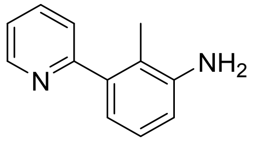	184.2	2.122	0.3	yes
TAH29	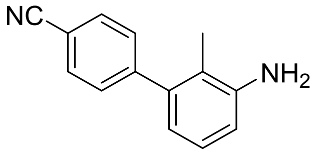	208.3	2.97	0.1	no
TAH30	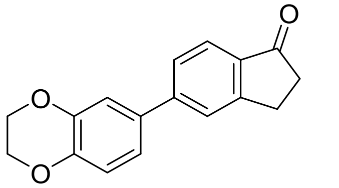	266.3	2.759	0.1	no
TAH34	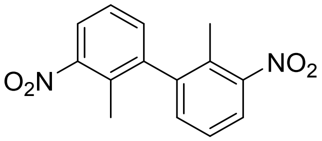	272.3	4.042	0.3	no
TAH35	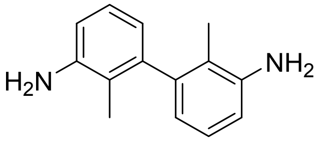	212.3	3.05	-1.2	yes
TAH36	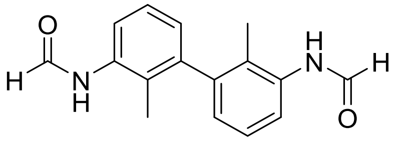	268.3	2.566	-1.4	yes
TAH37	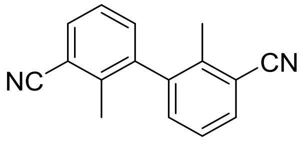	232.3	3.398	0.1	no
TAH38	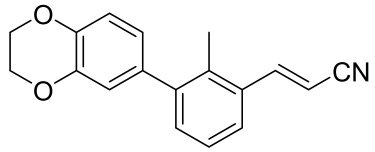	277.3	2.651	0.9	yes

TAH39	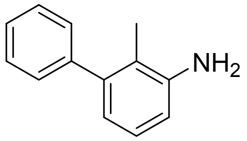	183.3	3.399	0.3	yes
TAH40	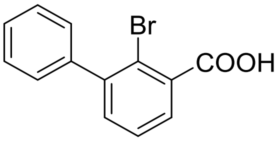	277.1	3.505	0.3	no
TAH41	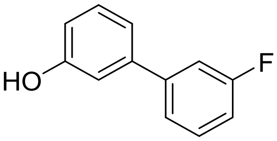	188.2	3.532	1.1	yes
TAH43	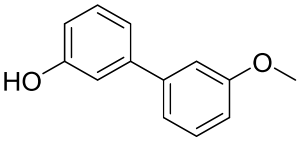	200.2	3.549	0.7	yes
TAH44	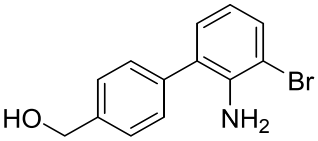	278.1	2.868	0.5	yes
TAH46	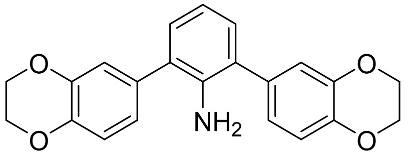	361.4	3.849	0.5	no
TAH47	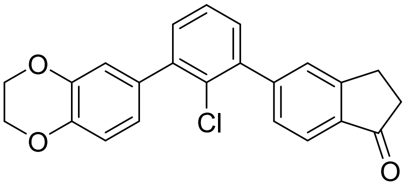	376.8	5.526	1.3	yes
TAH50	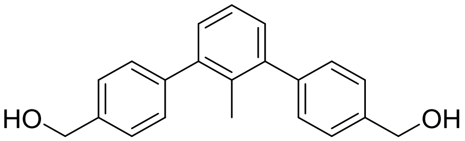	304.4	3.879	0.3	yes

TAH53	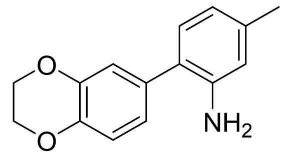	241.3	2.346	0.7	yes
TAH54	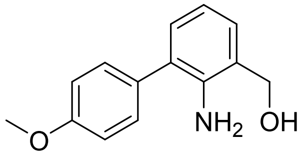	229.3	2.346	0.3	yes
TAH55	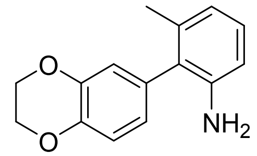	241.3	2.346	0.3	no
TAH56	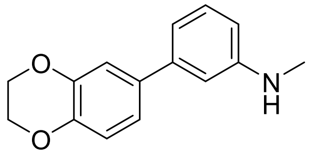	241.3	2.346	0.1	yes
TAH57	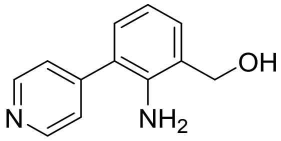	200.2	1.529	0.1	yes
TAH58	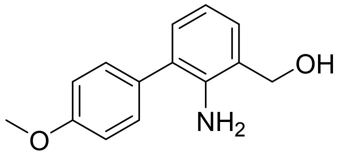	229.3	2.725	0.1	yes
TAH59	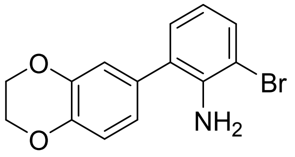	306.2	2.739	0.3	yes

TAH60	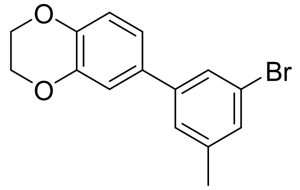	305.2	3.553	0.3	no
TAH61	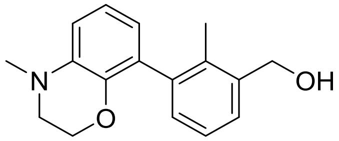	269.3	2.571	-1.1	yes
BMS-1166	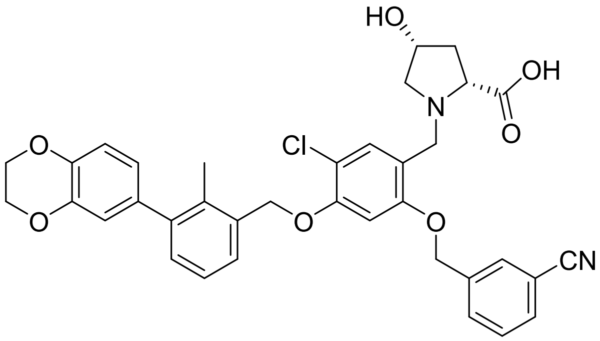	641.1	0.143	2.7	yes
STD4	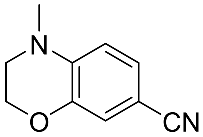	174.2	1.15	nt	yes

[a] – MW – molecular weight, MLogP – predicted logP using Moriguchi's model, both calculated in MedChemDesigner[1], nt - not tested

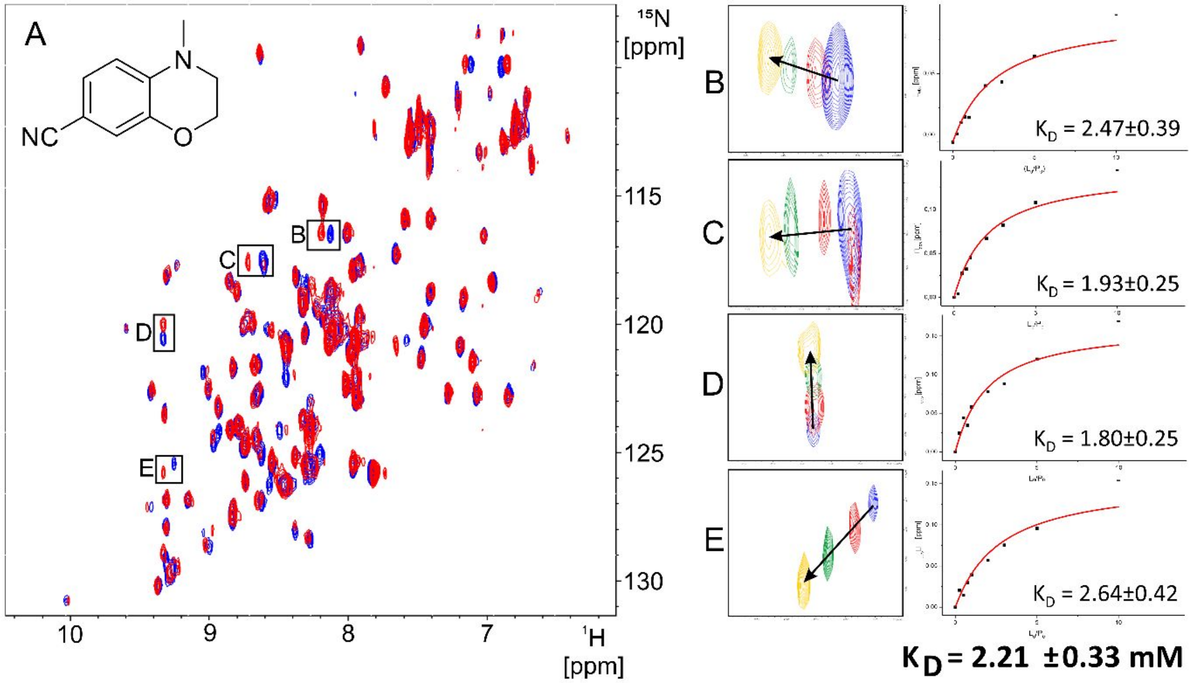

Figure S1. STD-4 binds to short PD-L1, A) Overlay of ^{1}H-^{15}N HSQC spectra of apo-short-PD-L1 (blue) and PD-L1 with **STD4** (red) at molar ratio 1:5. The individual cross-peaks perturbed by compound are marked with squares B-E; B-E) Sections of ^{15}N-^{1}H HSQC spectra of representative residues of the PD-L1 showing **STD4**-dependent chemical shift changes at **STD4**:PD-L1 molar ratio: 0:1 (blue contour), 1:1 (red contour), 1:5 (green contour), 1:10 (yellow contour) and binding curves derived from CSP of residues B-E with individual ('per residue') K_D values and average K_D derived from them

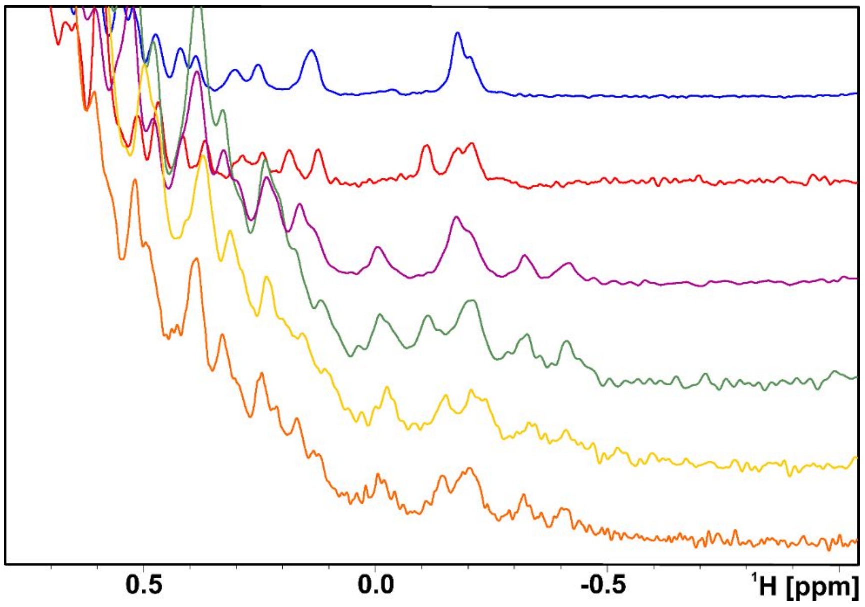

Figure S2. STD4, **TAH35** and **TAH36** bind to PD-L1 ectodomain; an aliphatic part of ^{1}H NMR spectra of the PD-L1 and small-molecule binders in molar ratio 1:10, respectively. References: short-PD-L1 (blue), short-PD-L1/**STD4** (red), references long-PD-L1 (purple), long-PD L1/**STD4** (green), long-PD-L1/**TAH35** (yellow), and long-PD-L1/**TAH36** (orange).

Table S2. List of probes used in FTMap analysis

#	Name	Abbreviation	Structure
1	Ethanol	EOL	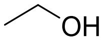
2	Isopropanol	THS	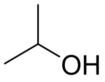
3	Isobutanol	BUT	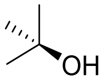
4	Acetone	ACT	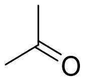
5	Acetaldehyde	ADY	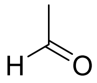
6	Dimethyl ether	DME	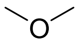
7	Cyclohexane	CHX	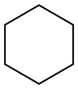
8	Ethane	ETH	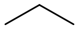

9	Acetonitrile	ACN	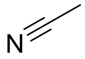
10	Urea	URE	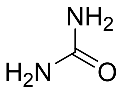
11	Methylamine	AMN	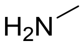
12	Phenol	PHN	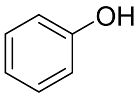
13	Benzaldehyde	BDY	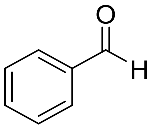
14	Benzene	BEN	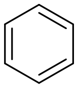
15	Acetamide	ACD	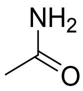
16	N,N-Dimethylformamide	DFO	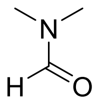

Supplementary References:

(1) MedChem Designer Version 4.5.0.12 (Simulations Plus Inc. Lancaster, CA), 2019
